# Tuberculosis Outbreak in a State Prison System — Washington, 2021–2022

**DOI:** 10.15585/mmwr.mm7212a3

**Published:** 2023-03-24

**Authors:** Randy M. Stalter, Monica Pecha, Lana Dov, David Miller, Zainab Ghazal, Jonathan Wortham, Sandy Althomsons, Molly Deutsch-Feldman, Rebekah Stewart, Derrick Felix, Sophia Hsu, Lara B. Strick

**Affiliations:** ^1^Epidemic Intelligence Service, CDC; ^2^Washington State Department of Health; ^3^Washington State Department of Corrections; ^4^Division of Tuberculosis Elimination, National Center for HIV, Viral Hepatitis, STD, and TB Prevention, CDC; ^5^University of Washington, Seattle, Washington.

During 2014–2020, no tuberculosis (TB) cases were reported within the Washington state prison system. However, during July 2021–June 2022, 25 TB cases were reported among persons incarcerated or formerly incarcerated in two Washington state prisons. Phylogenetic analyses of whole genome sequencing data indicated that *Mycobacterium tuberculosis* isolates from all 11 patients with culture-confirmed TB were closely related, suggesting that these cases represented a single outbreak. The median infectious period for 12 patients who were considered likely contagious was 170 days. As of November 15, 2022, the Washington State Department of Corrections (WADOC) and Washington State Department of Health (WADOH), with technical assistance from CDC, had identified 3,075 contacts among incarcerated residents and staff members at five state prisons, and 244 contacts without a known TB history received a diagnosis of latent TB infection (LTBI). Persons who were evaluated for TB disease were isolated; those receiving a diagnosis of TB then initiated antituberculosis therapy. Persons with LTBI were offered treatment to prevent progression to TB disease. This ongoing TB outbreak is the largest in Washington in 20 years. Suspension of annual TB screening while limited resources were redirected toward the COVID-19 response resulted in delayed case detection that facilitated TB transmission. In addition, fear of isolation might discourage residents and staff members from reporting symptoms, which likely also leads to delayed TB diagnoses. Continued close collaboration between WADOC and WADOH is needed to end this outbreak and prevent future outbreaks.

## Investigation and Results

During July–August 2021, one incarcerated person with TB disease and two others with LTBI were identified in a single Washington state prison (facility A). A subsequent source investigation conducted by WADOC in collaboration with WADOH identified one additional person at facility A with TB disease and 27 persons with LTBI. None of the persons who received a diagnosis during July–August 2021 experienced clinical characteristics associated with infectiousness (e.g., sputum smear positivity). This finding led to concern by WADOH and WADOC that these cases might represent transmission associated with a person with undiagnosed infectious TB disease elsewhere within the prison system or who was recently released from prison. During December 2021–January 2022, three persons incarcerated at another Washington state prison (facility B) received a diagnosis of TB disease, including a person who had been released into the community and another who had been transferred to a third facility (facility C). WADOC and WADOH requested CDC assistance to facilitate ongoing outbreak investigation efforts; CDC deployed a team to Washington on February 7, 2022. Outbreak cases, defined as clinically diagnosed or laboratory-confirmed pulmonary or extrapulmonary TB disease in persons who were incarcerated or had worked in WADOC since September 2019, were identified through facility-based testing and medical evaluations. Clinical chart reviews and provider interviews were used to characterize cases and estimate infectious periods according to CDC guidelines (*1*). Because of staff member and resident movement among prison facilities as well as releases into the community, the ongoing investigation has thus far included all 12 WADOC prisons and most of Washington’s local health jurisdictions. This activity was reviewed by CDC and was conducted consistent with applicable federal law and CDC policy.*

As of November 15, 2022, a total of 25 cases^†^ of TB disease among incarcerated persons had been reported to WADOH and were connected to the outbreak; the most recent case was reported on June 23, 2022 (Figure). No cases of TB disease were identified among prison staff members. Nineteen persons received a diagnosis of pulmonary TB disease with or without extrapulmonary TB. All 25 patients received a chest radiograph or computed tomography scan, and their sputum specimens were tested; none had cavitary findings on imaging, and four had a positive acid-fast bacilli smear. Isolates from all 11 culture-confirmed cases were closely related by whole genome single nucleotide polymorphism analysis, consistent with epidemiologic data suggesting recent transmission. Contact investigations were initiated at five facilities for 12 persons with TB disease who were considered likely to be contagious: the 11 persons with positive cultures and one additional person with a negative culture but with symptomatic pulmonary disease and clinically important chest radiograph findings. The median estimated infectious period for these 12 patients was 170 days (range = 91–391 days). The person with the longest infectious period was likely the first to develop infectious TB (i.e., estimated infectious period start date was January 1, 2021) and resided in facilities A and B during the infectious period. During the initial source investigation in August 2021, this person received a negative tuberculin skin test (TST) result and did not disclose symptoms, despite chart notes indicating a chronic cough and weight loss that began around July 2021. The person had a history of untreated LTBI, but this was not noted at the time. Therefore, a chest radiograph was not performed until a subsequent TB screening was conducted at facility B in January 2022; the radiograph findings were then reported to be abnormal.

**FIGURE:**
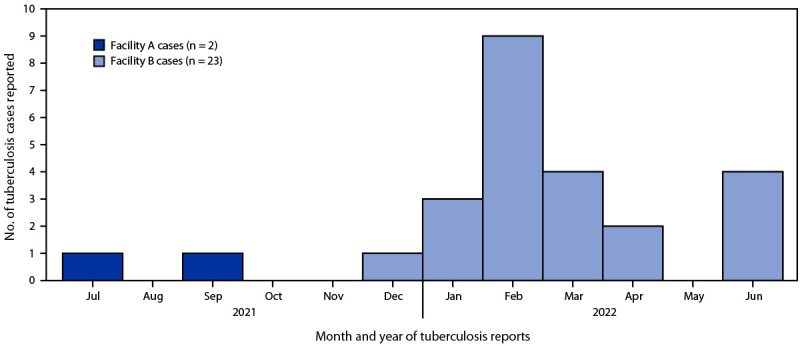
Outbreak-related tuberculosis cases reported by Washington State Department of Corrections to the Washington State Department of Health among persons who were incarcerated at two facilities, by month — Washington, July 2021–June 2022

A contact of a patient with TB was defined as an incarcerated person or staff member who had been in the same location on the same day as the patient during the patient’s estimated infectious period. As of November 15, 2022, a total of 2,644 residents in five facilities and 431 staff members in four facilities were identified as contacts. Among 2,093 (79.2%) resident-contacts and 135 (31.3%) staff-member–contacts who had no evidence of previous TB infection or disease and were tested within a WADOC facility since January 1, 2021, 237 (11.3%), and seven (5.2%), respectively, received positive TB test results.

## Public Health Response

After confirming the initial cases identified at facility A (in July 2021) and facility B (in December 2021), WADOC, in collaboration with WADOH, initiated TB screenings of incarcerated persons and staff members within facilities A, B, and C. Beginning in February 2022, CDC’s technical assistance team provided additional support for contact investigations by incorporating WADOC data on overnight locations and daily movements, staff member schedules, and clinical risk factors. Most persons born in the United States received testing using TSTs, and most persons not born in the United States received testing using interferon-gamma release assays. Following CDC guidelines for TB exposure, a TST result with ≥5 mm of induration in a specimen from a contact was considered positive (*1*). Persons with newly identified TB infection and persons with a previous positive TB test result or TB symptoms (irrespective of test result) were referred for chest radiography and clinical evaluation for TB disease. Persons who were being evaluated for TB disease were isolated and, if they received a TB diagnosis, initiated 4–6 months of antituberculosis therapy. For persons with LTBI, a 3-month isoniazid and rifapentine therapy was the preferred regimen. However, because of nationwide shortages of rifamycins, treatment was delayed for some persons; those persons who were considerably immunosuppressed from a medical condition or medication use were offered a 9-month isoniazid regimen to prevent delays in treatment because of their increased risk for progression to TB disease. Informational sessions on TB prevention and treatment were held for residents, their families, and facility personnel.

## Discussion

This is the first recorded TB outbreak in WADOC and the largest TB outbreak in Washington in 20 years. Multiple factors complicated case diagnosis and likely contributed to outbreak-associated transmission. First, annual TB testing of residents had been suspended at WADOC facilities, in some instances for up to 2 years, as WADOC redirected resources toward COVID-19 prevention and control. Although TB outbreaks in state prison systems before the COVID-19 pandemic had become uncommon (*2*), and WADOC had had no TB cases for approximately 5 years, these findings suggest that interruptions in routine TB prevention measures can facilitate *M. tuberculosis* transmission within correctional settings. Second, diagnostic delays contributed to outbreak-associated transmission because patients were contagious for longer periods; on-site and community clinicians did not promptly diagnose TB in two patients who were later found to have pulmonary TB disease, despite their having compatible symptoms. One of these patients was the person who had been transferred from facility A to facility B while contagious. Delayed detection of TB cases in low-incidence settings is a frequent contributor to outbreaks in the United States (*3*,*4*). The relative rarity of TB disease before the outbreak, the overlap of common TB symptoms with those of COVID-19, and a coincident COVID-19 outbreak within the prison system might have also contributed (*5*). In addition, fear of physical and social isolation among residents and potential social isolation and loss of work hours for staff members were likely disincentives to reporting symptoms or consenting to TB testing once screenings were initiated for many persons.

Outbreak response requires prompt diagnosis of TB disease, isolation of contagious persons, treatment of disease to cure, and prevention of disease through treating LTBI (*6*–*8*). During the outbreak response, nationwide shortages of rifamycins (*9*), cornerstones of preferred LTBI treatment regimens, led to delays in treatment initiation for some persons. During these shortages, alternative isoniazid monotherapy LTBI treatment regimens were prescribed only for persons at high risk for TB progression, because these regimens are longer in duration and are associated with increased risk for liver toxicity (*8*,*10*). Within WADOC, fully reinstating routine screening for TB symptoms and testing for LTBI and TB disease, raising TB awareness among incarcerated persons, staff members, and medical personnel, and implementing policies to reinforce symptom reporting and TB testing could facilitate earlier detection and intervention. In addition, establishing and maintaining efficient data management systems is important for managing contact investigations of this scale, actions for which state prisons are not always equipped or funded. Therefore, ongoing strong collaborations between correctional systems and health departments are needed to end this outbreak and prevent future outbreaks.

**Summary**

**What is already known about this topic?**

Tuberculosis (TB) outbreaks in state prisons are uncommon. During 2014–2020, no TB cases were reported within the Washington state prison system.

**What is added by this report?**

During 2021–2022, a total of 25 TB cases were reported among persons incarcerated in two Washington state prisons. An additional 244 resident-contacts and staff-member–contacts without known TB histories in five facilities received a diagnosis of latent TB infection.

**What are the implications for public health practice?**

This is Washington’s largest TB outbreak in 20 years. Transmission was facilitated by prolonged case infectiousness and suspension of annual screenings because clinical resources were diverted to the COVID-19 pandemic response. Close collaborations between corrections departments and public health officials will be critical for ending this outbreak and preventing future TB outbreaks.
